# Induction of Epithelial–Mesenchymal Transition in Periodontitis Rat Model

**DOI:** 10.1055/s-0044-1792011

**Published:** 2024-12-30

**Authors:** Basma F. Alanbari, Firas B. Al-Taweel, Paul R. Cooper, Mike R. Milward

**Affiliations:** 1 Department of Periodontics, College of Dentistry, University of Baghdad, Bab Al Mudam, Baghdad, Iraq; 2 Department of Dentistry, Periodontics Branch, Al-Rafidain University College, Baghdad, Iraq; 3 Faculty of Dentistry, Sir John Walsh Research Institute, University of Otago, Dunedin, New Zealand; 4 School of Dentistry, University of Birmingham, Birmingham, United Kingdom

**Keywords:** animals, alveolar bone loss, biomarkers, cadherins, epithelial–mesenchymal transition, periodontitis, vimentin

## Abstract

**Objectives** Epithelial–mesenchymal transition (EMT) is a process that shifts cellular phenotype. It is linked to several different inflammatory diseases including periodontitis. This study was conducted to investigate the involvement of the EMT process in an experimental periodontitis (EP) model.

**Materials and Methods** Second upper molars of Wistar albino male rats were ligated to induce periodontitis, while controls were not ligated. The animals were sacrificed after 0, 3, 7, 14, and 21 days ( *n* = 6 for each time point). The maxillae were resected, posterior to the incisor teeth, and the gingival tissue surrounding teeth were analyzed. Alveolar bone loss (ABL), epithelial thickness, and the number of inflammatory cells were measured at each time point. Expressions of EMT-related biomarkers (E-cadherin, N-cadherin, Snail1, Twist1, and vimentin) were assessed using the immunohistochemical technique. All experiments were performed in triplicate.

**Statistical Analysis** Inferential comparisons were performed by the kruskall-wallis test. To determine the correlation between the dependent and independent variables ,Spearman's correlation test was used.

**Results** ABL, epithelial thickness, and inflammatory cell count were gradually increased throughout the EP study period. Switching of E-cadherin/N-cadherin was evident and associated with increased nuclear expression of Snail1 and Twist1. Additionally, positive cytoplasmic expression of vimentin was detected from day 7 and increased at subsequent time points. Histoscore of E-cadherin was negatively and significantly correlated with N-cadherin and Snail1. Furthermore, Snail1 and Twist1 histoscores were significantly and positively correlated.

**Conclusion** The results demonstrated induction of an EMT phenotype in the EP model. This was supported by cadherin switching and positive vimentin expression along with nuclear translocation of Snail1 and Twist.

## Introduction

Periodontal disease is the sequalae of exaggerated immune response to the presence of dysbiotic microbiota on the tooth surface and within the gingival sulcus. 1 The epithelium provides the first anatomical and immunological barrier blocking the ingress of periodontal pathogens and their virulence factors to deeper tissues and systemic circulation. 2 Keratinocytes are interconnected by cellular junctions, which consist of special structural proteins. 3 Dissolution of those junctional proteins compromise the epithelial barrier, providing a direct route for bacterial ingress into the periodontal connective tissue resulting in tissue breakdown. 4 Epithelial–mesenchymal transition (EMT) is a process leading to phenotype shifting from an epithelial to a mesenchymal phenotype. It is defined by key sequential events including the dissolution of the epithelial junctions, loss of apical–basal polarity with acquisition of front–rear polarity, reorganization of the cytoskeleton, downregulation of epithelial genes and proteins, and upregulation of their counterparts, which define the mesenchymal phenotype. Consequently, cells exhibit increased motility, and acquire invasive characteristics that induce extracellular matrix degradation. 5 EMT has been extensively studied during embryonic development, malignancy, and tissue fibrosis. Evidence from several *in vitro* studies now confirms that EMT is induced by periodontal pathogens, particularly gram-negative species, such as *Porphyromonas gingivalis* , *Fusobacterium nucleatum* , *Treponema denticola* , and *Aggregatibacter actinomycetemcomitans* . 6 7

E-cadherin-dependent adherens junctions are a prerequisite for the functional assembly of other intercellular junctions including tight junctions and desmosomes. 8 Epithelial cells undergoing EMT are typically characterized by a shift in their cadherins from E-cadherin to N-cadherin, and partial or complete replacement of keratin with vimentin. 5 The EMT regulatory consortium comprises four different elements: transcriptional factors (TFs), small noncoding RNAs, differential splicing, as well as translation and posttranslational regulators. EMT key TFs include Snail and Twist, which are best characterized within the context of tissue development, fibrosis, and cancer. Reported results from *in vitro* and *in vivo* studies have documented their role in activating EMT programs in epithelial cells in both developmental and pathological conditions. 5 9 Snail and Twist exhibit unique expression profiles induced by a variety of microbial, inflammatory, and ecological stressors. 10 11 In EMT, Snail and Twist collaboratively or separately suppress E-cadherin expression and simultaneously induce N-cadherin expression. 12 Additionally, the motility and invasive potential of the transitioned cells are enhanced by increased vimentin expression induced by these TFs. 13 Furthermore, as part of a regulatory loop, Snail and Twist enhance the expression of each other, and act synergistically on their targeted genes, including several other EMT-TFs. 12 14

To date, no studies have explored the expression of EMT key biomarkers in experimental periodontitis (EP) models. Consequently, this study was conducted to investigate the EMT phenotype expression within this context.

## Materials and Methods

### Animals and Ethical Approval

**Fig. 1:**
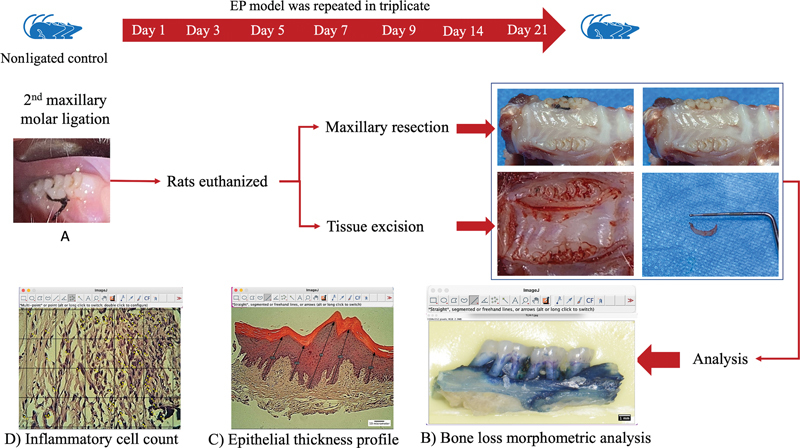
Design of ligature-induced periodontitis pilot study. (Top) For the pilot study, experimental animals were allocated into two groups: ligation (L) and unligated control (3 animals) . The animals in the ligation group (21 animal) were subdivided into 7 groups corresponding to experimental periodontitis' induction timelines 1, 3, 5, 7, 9, 14, and 21 days' timelines. ( **A** ) Representative clinical image of the ligated molar tooth, After sacrificing the rats, the maxilla was resected to prepare bone and soft tissue for analysis. ( **B** ) The analyses included measurement of alveolar bone loss by morphometric analysis from the cementoenamel junction to the alveolar bone crest (black lines), ( **C** ) measuring the thickness of the epithelium, and ( **D** ) counting the number of inflammatory cells. EP, experimental periodontitis.

The experimental protocol for surgical procedures and animal treatment was approved by the Ethics Committee, College of Dentistry, University of Baghdad (Project no. 65122 on September 13, 2022). All animals received human care according to the Animal Research: Reporting of *In Vivo* Experiments (ARRIVE) guidelines. 15 A pilot study was conducted to identify the TLs analogous to different stages of periodontitis. EP was induced in 24 animals for 21 days, 3 animals were scarified at days 1, 3, 5, 7, 9, 14, and 21 ( Fig. 1 ). According to the results of the pilot study, days 3, 7, 14, and 21 were selected in this study representing the periodontitis terminal phases of stages 1, 2, 3, and 4, respectively.

Sample size was calculated using resource equation approach 16 :

*n* = *DF* / *K* + 1,

where *DF* stands for the degree of freedom, *K* for the number of groups (5 experimental timelines including control), and *n* for the number of animals per group. Since the acceptable range of *DF* is 10 as the minimum and 20 as the maximum, accordingly the minimum and maximum *n* were calculated as follows:

Minimum *n* = 10/ *K* + 1.

Maximum *n* = 20/ *K* + 1.

Accordingly, the maximum number required was 5 animals/timeline. Taking into account 10% as attrition rate, the total number was 6 animals/timeline. For this study, a total of 30 Wistar albino male rats aged 2 months, disease free with an average body weight of 250 to 300 g, were purchased from Al Dhya'a Advanced Pet Hospital, Baghdad, Iraq. The rats were kept in the animal facility under standardized conditions: humidity at 60%, temperature controlled at 22 ± 2°C, and a 12-hour light/dark cycle. The rats acclimated for 1 week prior to the first procedure within plastic cages identified by their number, group, and date. During the entire experimental period, animal monitoring was undertaken daily.

### Ligature-Induced Experimental Periodontitis

Simple randomization was used to assign rats into nonligated controls and EP timeline groups. The animals were anesthetized using intramuscular (IM) injection, mixture of 10% ketamine hydrochloride (80 mg/kg) and 2% xylazine hydrochloride (10 mg/kg), with a dose of 0.05 ml/kg of body weight intraperitoneally. Full anesthesia set in within 3 to 5 minutes and was confirmed by the absence of response when the rat toe was pinched. To induce bone loss, a 5–0 silk ligature was tied around the right maxillary second molar, as described by Pereira SSC et al. ( Fig. 1A ). 17 Ligatures were checked daily throughout the experimental period. All rats were euthanized humanely at the end of treatment under general anesthesia overdose. To ensure the animals were dead, a secondary method, cervical dislocation, was used. A pilot study was initially conducted to confirm the induction of EP and to characterize TLs reflecting the severity of bone loss corresponding to clinical stages of periodontitis in humans. All experiments were repeated in triplicate both technically and biologically ( Fig. 1 ).

### Sample Preparation

The maxillae were resected and bisected from both sides, posterior to the incisor teeth. The gingival tissue around ligated teeth was sampled from the resected maxillae using a no. 11 scalpel blade by an initial horizontal shallow sulcular incision around the maxillary molars, avoiding deep cutting as this point may cause the thin maxilla to sever. The second horizontal incision was made parallel to the first near the palatal midline. Both incisions were joined by two vertical incisions. The entire gingival band was carefully removed by blunt dissection. The collected samples were cleaned by tap water and immediately stored in plastic containers containing 10% neutral buffered formalin solution for tissue fixation for 24 hours prior to further processing and histological analysis ( Fig. 1 ).

### Bone Loss Morphometric Analysis

The hemimaxillae were fixed in formalin for 24 hours prior to mechanical defleshing. To facilitate removal of the excess tissue, the hemimaxillae were immersed in 5% sodium hypochlorite for 3 hours. Specimens were cleaned under running water and air dried at room temperature. To enhance cementoenamel junction (CEJ) visualization, the specimens were stained for 1 minute with 1% methylene blue. 18 For standardization, the specimens were fitted in a mold made of heavy body impression putty within a hard paper frame with their palatal side facing upward and the occlusal plane parallel to the surface below. The long axis of each specimen was aligned perpendicular to the camera, and a millimeter-scale ruler fitted in the mold used as a reference, prior to digital photographic documentation. Standardized digital photography was used to capture images of the specimens stained with methylene blue using macro lens (105 mm, Sigma, Japan), with a 1:1 ratio, mounted on a digital camera (Nikon D7200, 24.2 megapixels, Japan). A calibrated examiner performed the morphometric analysis using ImageJ Software (NIH, United States). The distance from the CEJ to the alveolar bone crest (ABC) on the palatal face of the maxillary second molar was measured. Three points were measured for every specimen: one in the center of the mesial and distal root and one in the furcation area, and the mean of these reading was then computed ( Fig. 1B ).

#### Epithelial Thickness

The gingival samples were longitudinally trimmed (mesiodistal direction) and routinely prepared to obtain hematoxylin and eosin (H&E) tissue sections. Microscopic images for all H&E slides of EP TLs were captured under ×10 magnification (Leica Qwin 500, Wetzlar, Germany) using a digital video camera and an image analysis system (Motic, ToupTek, ToupView, ×86, 3.7.4183, and 2014; Fig. 1C ). The epithelial thickness of each EP TLs was determined by averaging the epithelial thickness. The change in epithelial thickness was calculated as the difference between the nonligated control and EP TLs.

### Inflammatory Cell Count

For all H&E slides, quantitative assessment for inflammatory infiltrate in the gingival connective tissue at the palatal side was performed under ×40 magnification by calibrated examiners. Images were evaluated for inflammation intensity using multipoint tool of ImageJ software. Five microscopic areas were randomly selected, and each field was divided into 20 squares by an ImageJ grid tool ( Fig. 1D ). The mean count of inflammatory cells was determined and classified as follows: score 0 (0–25 cells), score 1 (26–50 cells), score 2 (51–75 cells), and score 3 (>75 cells). 19

### Immunohistochemical Staining

Fixed samples were dehydrated in ascending grades of alcohol, cleared in xylol, embedded in paraffin blocks. From each paraffin-embedded tissue block, serial sections, 4-μm thickness, were dissected in the mesiodistal direction and mounted on positively charged slides (Fisher Scientific and Escho superfrost plus, United States) for immunohistochemical staining (IHS). The following antibodies were used: monoclonal anti-E-cadherin (1:200, E-AB-70249), polyclonal anti-N-cadherin (1:300, E-AB-70061), polyclonal antivimentin (1:200, E-AB-70081), polyclonal anti-Snail (1:100, E-AB-65888), and polyclonal anti-Twist (1:200, E-AB-66494). The antigen–antibody binding signal was amplified by 2-step plus Poly-HRP Anti Rabbit/Mouse IgG Detection System with DAB solution (E-IR-R217). All products were purchased from Elabscience Biotechnology Inc., United States. Positive controls were obtained from the tissues recommended by the manufacturer, while negative controls for all biomarkers were prepared by adding phosphate buffered saline (PBS) instead of the primary antibodies ( Supplementary Fig. 1 , available in the online version only).

#### Immunohistochemical Scoring

Digital images of IHC-stained slides were evaluated by the main investigator, and blindly calibrated with an oral pathologist until an agreement ≥90% for the histoscore was achieved. A semiquantitative approach was used to grade the immunoreactivity for membranous staining of E-cadherin, N-cadherin, Snail, and Twist. These were measured using the following scale: –, negative expression; +, weak expression; ++, moderate expression; and +++, strong expression. The percentage of cells stained at each intensity level was graded as 0 (<5%), 1 (5 ± 25%), 2 (26 ± 50%), 3 (51 ± 75%), and 4 (>75%). Both were multiplied to calculate the final histoscores as previously described. 20 21 For vimentin, the presence/absence of cytoplasmic expression was recorded as a dichotomous score (1, 0). 22 23

### Statistical Analysis

GraphPad Prism Software (Version9.0; La Jolla, CA, United States) was used to analyze the raw data. Both parametric and nonparametric distribution of the datasets was verified using the Shapiro–Wilk test. Accordingly, inferential comparisons were performed by the Kruskal–Wallis test. To determine the correlation between the dependent and independent variables, Spearman's correlation test was used. The level of significant difference was determined when *p* < 0.05.

## Results

### Morphometric Analysis of Alveolar Bone Loss

**Fig. 2:**
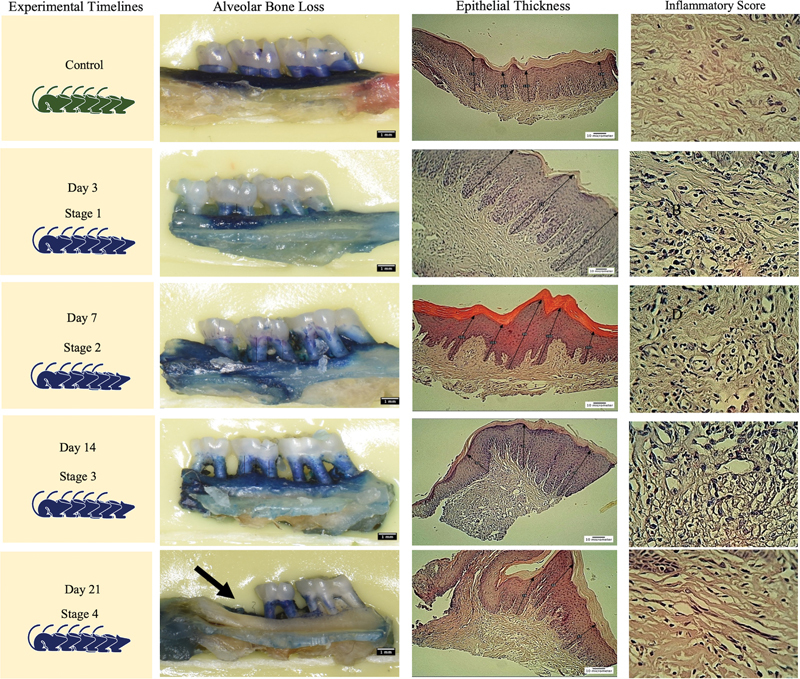
Left panel: Morphometric analysis showed that bone loss was 15% at day 3, 33% at day 7, greater than 33% at day 14, and greater than 33% at day 21, associated with tooth loss ( *arrow* ). Middle panel: Representative photomicrographs of the gingival samples. Control and day 3 showing mild elongation and normal configuration of the epithelial ridges. In contrast, days 7, 14, and 21 showed marked rete peg elongation and epithelial hyperplasia. Note the tortuous appearance of the epithelial ridges. Hematoxylin and eosin (H&E) stain, ×10 magnification. Right panel: Photomicrographs of the gingival samples showing the gradual increase the number of inflammatory cell counts. Note the marked reduction in inflammatory cell counts observed on day 21. H&E stain, ×40 magnification.

**Fig. 3:**
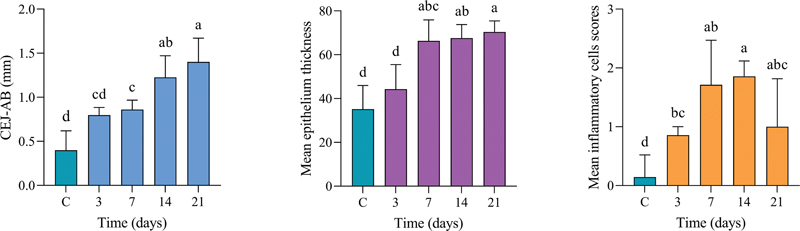
Left: Significant alveolar bone loss was observed at day 7 in comparison with control (C), which continued to days 14 and 21. Middle: Epithelium was significantly thicker at days 7 to 21 in comparison with day 3 and control (C). Right: Inflammatory infiltrates were significantly higher at all time points than control (C). Significant differences were at *p* < 0.05 using the Kruskal–Wallis test. The superscript letter “a” represents the highest value, while shared letters indicate nonsignificant differences. AB, alveolar bone; CEJ, cementoenamel junction.

The alveolar bone loss (ABL) observed in all representative TLs was significantly different from controls except for day 3 ( Fig. 2 ). The significant difference of ABL was evident between successive TLs till day 14, which was not significantly different from day 21 ( Fig. 3 , left panel).

### Epithelial Thickness

The gingival epithelial thickness showed a marked increased thickening in the EP model ( Fig. 2 ). The gingival epithelium at days 14 and 21 displayed pronounced epithelial hyperplasia and were significantly different from controls. Elongated epithelial ridges extending deeply into the underlying lamina propria were observed during days 7, 14, and 21. In contrast, epithelial ridges at day 3 resembled those in the control group ( Fig. 3 , middle panel).

### Inflammatory Cell Counts

All EP-TLs displayed higher inflammatory scores when compared with the nonligated control ( Fig. 2 ). The mean inflammatory cell count showed a gradual increase beginning from the first day postligation and continued to peak at day 14, followed by a decline at the end of the experimental period. Significantly, there were lower inflammatory cell counts in controls compared with days 14 and 21 ( Fig. 3 , left panel).

### Immunohistochemical Expression of EMT Markers

#### E-Cadherin to N-Cadherin Switch

**Fig. 4:**
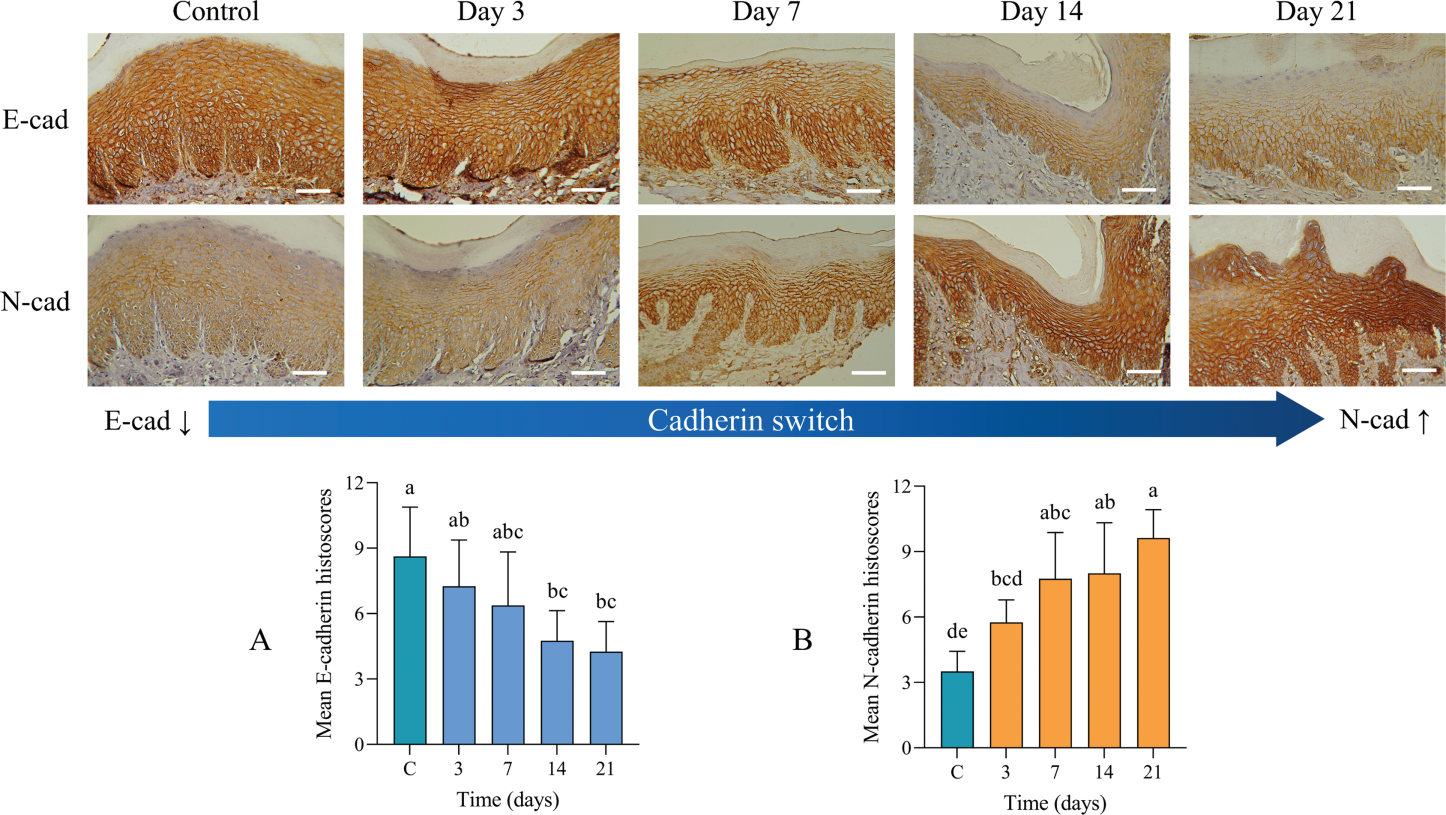
Cadherin switching in the experimental periodontitis model. ( **A** ) Immunohistochemical (IHC) staining showed that E-cadherin (E-cad) IHC scores were decreased significantly at days 14 and 21 compared with control (C). ( **B** ) Concomitantly, N-cadherin (N-cad) IHC scores were significantly increased at days 7, 14, and 21 compared with day 3 and control (C). Significant differences were identified at *p* < 0.05 using the Kruskal–Wallis test. The superscript letter “a” represents the highest value, while shared letters indicate non-significant differences. Scale bar 80µm.

IHC analysis demonstrated that the E-cadherin histoscore was strongly expressed in control and days 3 and 7, and declined at days 14 and 21. Inferential analysis showed significantly higher E-cadherin histoscore in controls as compared with days 14 and 21. In addition, the E-cadherin expression was significantly lower at day 21 than its expression at day 3. In contrast to N-cadherin, data showed constant upregulation and the highest N-cadherin histoscore was observed at day 21, which was significantly different from controls ( Fig. 4 ).

#### EMT-Transcriptional Factors (Snail and Twist)

**Fig. 5:**
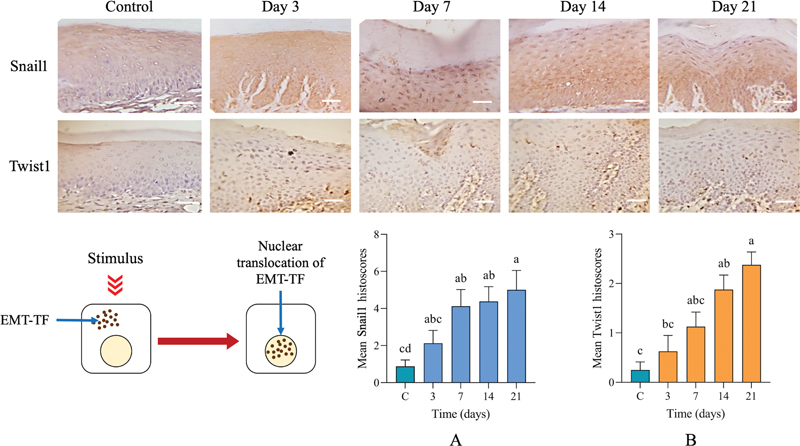
Immunohistochemical (IHC) staining of epithelial–mesenchymal transition-transcriptional factors (Snail1 and Twist1). Nuclear IHC expression of both transcriptional factors was significantly increased toward the end of the experimental period. Significant differences from control (C) appeared earlier with ( **A** ) Snail1, at day 3, ( **B** ) while Twist1 showed significant difference at day 7. Significant differences at *p* < 0.05 using the Kruskal–Wallis test. The superscript letter “a” represents the highest value, while shared letters indicate nonsignificant differences. Scale bar: 80 µm.

IHC images showed that the positive expression of nuclear EMT-TFs, Snail1 and Twist, gradually increased throughout the EP period ( Fig. 3 ). Snail1 histoscores were significantly higher after 7, 14, and 21 days postligation in comparison with controls. Twist histoscores showed similar patterns to Snail1 except that significantly higher differences from control were not observed until days 14 and 21 ( Fig. 5 ).

#### Vimentin

**Fig. 6:**
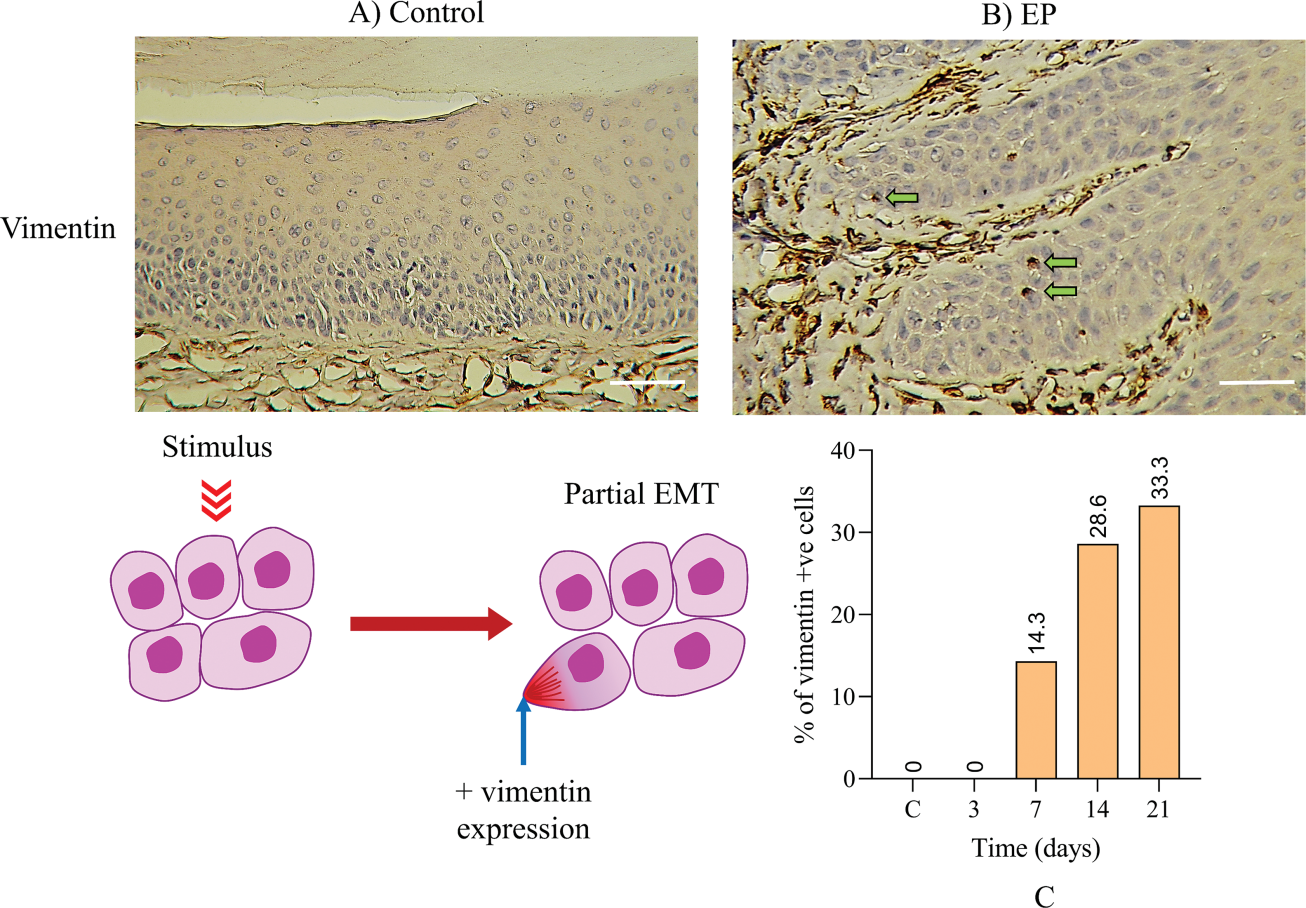
Immunohistochemical (IHC) staining for vimentin. ( **A** ) Nonligated controls exhibited negative vimentin expression, ( **B** ) while positive cytoplasmic IHC expression of vimentin ( *green arrows* ) was observed at day 7 in 14.3% of cell populations, ( **C** ) which gradually increased to 33.3% at day 21. Scale bar: 100 µm. EMT, epithelial–mesenchymal transition; EP, experimental periodontitis.

Analyses showed that both control and the day 3 group did not exhibit positive cytoplasmic expression of vimentin ( Fig. 4 ). Positive expression was initially evident at day 7 in few discrete cells (14.3%) at the basal layer of the epithelium adjacent to the basement membrane, and a consistent trend of expression was evident at days 14 and 21 ( Fig. 6 ).

### Correlation of EMT Key Biomarkers and Transcriptional Factors

The IHC histoscores of E-cadherin exhibited a significant negative correlation with N-cadherin and Snail1. EMT-TFs, Snail1 and Twist, were significantly and positively correlated. In addition, vimentin showed nonsignificant positive and negative correlations with Twist and E-cadherin, respectively ( Table 1 ).

**Table 1:** Correlation of epithelial–mesenchymal transition histoscores

		E-cadherin	N-cadherin	Vimentin	Snail1	Twist
**E-cadherin**	*r*		–0.620	–0.13	–0.40	–0.105
	*p* value a		**<0.001**	0.41	**0.01**	0.51
**N-cadherin**	*r*	–0.616		0.046	0.223	0.178
	*p* value a	**<0.001**		0.77	0.16	0.27
**Vimentin**	*r*	–0.133	0.045		–0.070	0.258
	*p* value a	0.41	0.77		0.66	0.10
**Snail1**	*r*	–0.394	0.223	–0.070		0.481
	*p* value a	**0.02**	0.16	0.66		**0.002**
**Twist**	*r*	–0.105	0.178	0.258	0.481	
	*p* value a	0.51	0.27	0.10	**0.002**	

Note: *r* : Spearman's correlation coefficient.

a Significant correlation at *p* < 0.05 indicated by bold font.

## Discussion

The present study investigated the longitudinal IHC expression of EMT-key biomarkers in an EP model. Data confirmed the EMT-associated cadherin switch together with a positive expression of vimentin as well as upregulation of EMT-TFs, Snail1 and Twist. These novel results supported the potential role of EMT as a pathogenic mechanism involved in initiation and progression of periodontitis. The ligature EP is a common technique for driving bone resorption in animal studies, which is based on inducing dysbiosis of the microbiome mimicking that of periodontitis in a relatively short period. The maxillary second molar was selected for ligation due to faster bone resorption and more rapid onset of periodontitis as a result of the porosity of the maxilla. 24 25 Additionally, this site provides enhanced suture retention in the two interdental areas. 17

In this study, E-cadherin expression was decreased and associated with increased N-cadherin expression, a hallmark of EMT. 26 27 These changes will compromise the integrity of the gingival epithelial barrier, which is evident during periodontal disease initiation and progression. 11 22 28 This event is likely attributed to the microbial dysbiosis induced by the ligature as the literature indicates the role of these bacteria and their virulence factors in driving epithelial barrier function-related gene/protein expression via E-cadherin dysregulation. It has been shown that arginine- and lysine-specific gingipains of *P. gingivalis* , such as HRgpA, RgpB, and Kgp, have a hydrolytic effect on E-cadherin. 29 Challenging human gingival epithelial cells (HGEC) with *P. gingivalis* lipopolysaccharide ( *P. gingivalis* -LPS) leads to disruption of the epithelial barrier, which accelerated the penetration of *P. gingivalis-* LPS through the cell monolayer. 30 Whole live *A. actinomycetemcomitans* application to rat gingival sulcus and cultured HGEC decreased the level and expression of E-cadherin, zonula occludens-1, and connexin. Additionally, treating HGEC with recombinant Cdt of *A. actinomycetemcomitans* resulted in detachment of the keratinized outer layer, distention of spinous and basal cells in the oral epithelium, and disruption of the rete pegs. 31 Furthermore, *F. nucleatum* –encoded adhesion protein FadA binds to E-cadherin, inducing its phosphorylation on the membrane, internalization, and decreased expression.

EMT-TFs have key expression profiles, and their regulation of the EMT process targeted particular cells and tissues to activate signaling pathways for EMT induction. 32 This study has identified a positive correlation between Snail1 and Twist supporting their known role in repressing E-cadherin expression by directly and indirectly binding to its promoter. 33 Indeed, Saliem et al previously confirmed positive Snail1 expression accompanied by reduced E-cadherin levels in inflamed gingival tissues. 22 Moreover, Snail1 and Twist exhibit a dual role by repressing E-cadherin and inducing N-cadherin, indicating that the cadherin switch is key to the transcriptional reprograming process of EMT. 34 Notably, cadherin switching affects the epithelial barrier at multiple levels; N-cadherin-mediated adherens junction are weaker than E-cadherin junction, and consequently influence keratinocyte shifting from an adhesive epithelial state into a motile mesenchymal invasive state. This phenomenon is mediated by several signaling pathways including Wnt/β-catenin, PI3K/Akt, RhoA activation, and MAPK-Erk, along with expression of a wide range of proteolytic metalloproteinases. 35 The cleaving and proteolytic action of the latter facilitates downregulation and shedding of E-cadherin, which increase epithelial permeability, inducing basement membrane microulceration, and enable invasion of the connective tissue by periodontal pathogens. 36

Periodontal pathogens and/or their virulence factors are considered potent Snail1 inducers. 30 Challenging rat oral keratinocytes with *P. gingivalis* and *F. nucleatum* induced expression of Snail1 together with other EMT regulators. 11 Indeed, the inflammatory response induced by the periodontal dysbiotic microenvironment is enriched with a wide array of Snail1 inducers. 37 These are responsible for inducing complex downstream signaling networks affecting Snail1 expression, including receptor tyrosine kinases signaling, which act through the Ras-MAPK or PI3K-Akt, TGF-β, Notch, Wnt, NF-κB, TNF-α, and BMPs pathways Furthermore, posttranslational modifications such as Gsk3β-mediated phosphorylation of Snail1 are initiated by these aforementioned signaling pathways, thus controlling its cellular localization, stability, degradation, and activity. 38

Vimentin was previously identified in the human gingival crevicular fluid by quantitative proteomic analysis and was more closely associated with periodontitis compared with gingivitis and gingival health. 39 During EMT, cells shift their IF composition from being keratin dominant to vimentin dominant. Although both are responsible for trafficking organelles and membrane-associated proteins, they show different protein preferences. While keratins direct E-cadherin to the cellular membrane enabling cellular adhesion, vimentin interacts with motor proteins enabling cellular motility. 40 Our IHC findings revealed a gradual increase in the number of vimentin-positive cells subjacent to the basement membrane. These data were consistent with *in vitro* evidence of epithelial cells expressing vimentin upon exposure to periodontal pathogens. 11 In addition, positive vimentin expression in inflamed human tissue samples of periodontitis patient was also reported. 22 28 EMT is a multistage process and vimentin-positive cellular expression is associated with the advanced state. Current findings support this notion since the positive expression was observed at the advanced EP stage.

The latest guidelines recommend a multileveled investigational panel to confirm EMT, including EMT molecular markers along with changes in the cellular and behavior properties rather than relying on the sole influence of the molecular markers. In the current study, EMT was investigated in the rat inflamed gingival tissue samples utilizing only the immunohistochemical expression profile of EMT biomarker and TFs. Such limitation arise since this study was self-funded. Further panels of analysis were not applicable due to the limited laboratory and technical support as well as lack of institutional financial grants. 5 Currently, and, to the best of our knowledge, the results of this preclinical study are novel and demonstrate the positive expression of EMT biomarkers in EP. These findings could pave the way for future experimental studies and for exploring unidentified mechanistic links between periodontitis and EMT. Additionally, this model could provide an opportunity for testing EMT inhibitors preclinically as a novel approach to treat the disease.

## Conclusion

The results of this EP model demonstrated induction of an EMT phenotype in diseased periodontal tissue. This was confirmed by cadherin switching and positive vimentin expression along with nuclear translocation of Snail1 and Twist. Further studies are required to better characterize the induction of the EMT phenotype in the EP model and these data could then provide a platform to better understand disease progression and identify pharmaceuticals for disease management and treatment.
