# Supplementary material for: Induction of Epithelial–Mesenchymal Transition in Periodontitis Rat Model
Source: Eur J Dent. 2024 Dec 30;19(2):428–37. doi: 10.1055/s-0044-1792011 (PMC12020583; doi:10.1055/s-0044-1792011)
Supplement: Supplementary file 1 — Supplementary Material [file 10-1055-s-0044-1792011-s2433419.pdf]

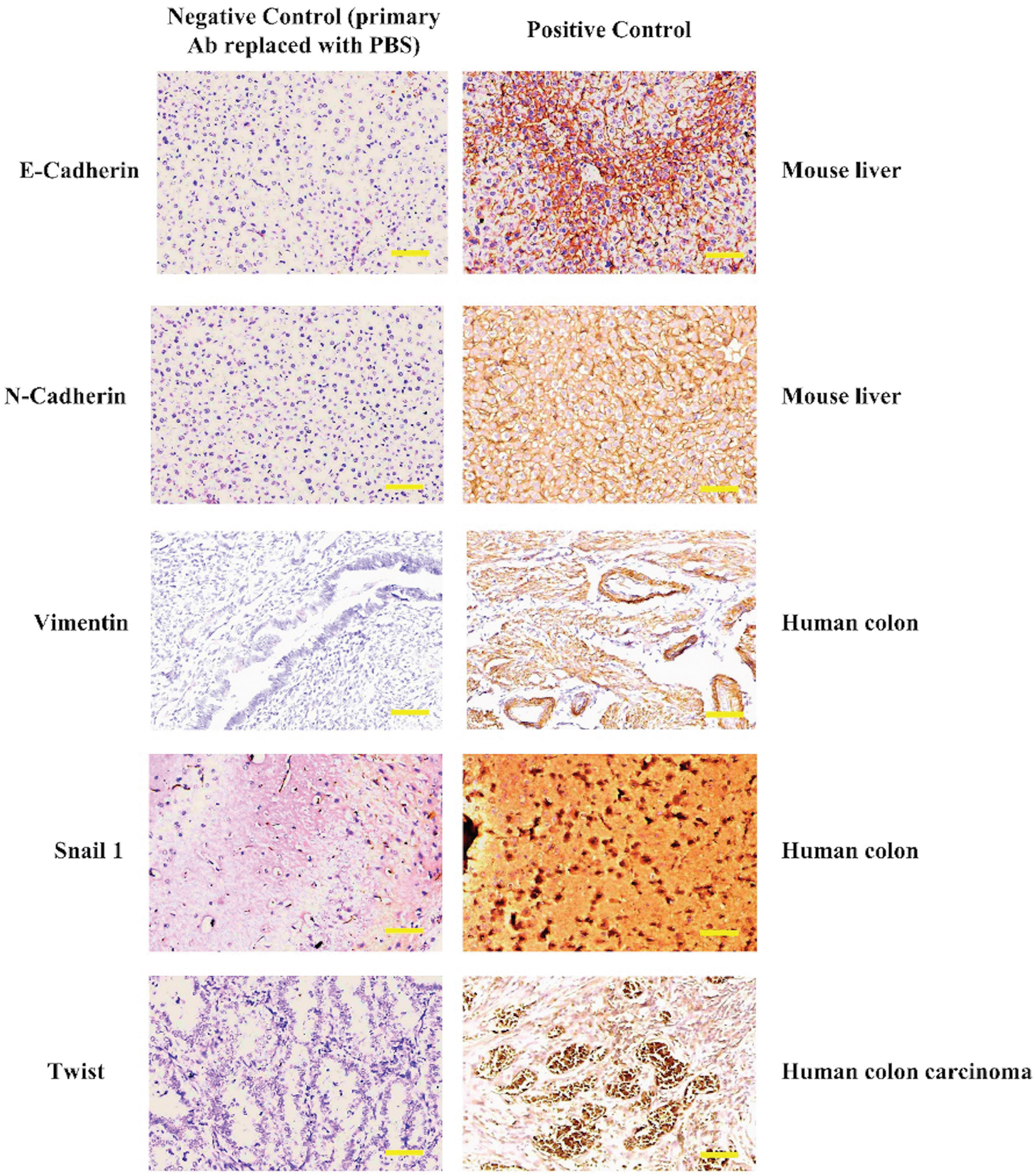

Supplementary Fig. S1 Positive and negative controls for the epithelial–mesenchymal transition biomarkers used in the present study. Scale bar: 30 µm. The positive immunoreactivity of the primary antibodies in positive tissue controls used in the study were as follow: E-cadherin and N-cadherin reactivity in mouse liver, vimentin reactivity in human colon, Snail1 reactivity in mouse brain, and Twist reactivity in human colon cancer. PBS, phosphate buffered saline.

 © 2024. The Author(s).
